# Cortisol levels in cerebrospinal fluid correlate with severity and bacterial origin of meningitis

**DOI:** 10.1186/cc5729

**Published:** 2007-03-27

**Authors:** Michal Holub, Ondřej Beran, Olga Džupová, Jarmila Hnyková, Zdenka Lacinová, Jana Příhodová, Bohumír Procházka, Miroslav Helcl

**Affiliations:** 1 3rd Department of Infectious and Tropical Diseases of First Faculty of Medicine, Charles University in Prague, Budínova 2, CZ-180 81, Prague, Czech Republic; 2 Department of Infectious Diseases, University Hospital Bulovka, Budínova 2, CZ-180 81, Prague, Czech Republic; 3 Department of Infectious Diseases of Third Faculty of Medicine, Charles University in Prague, Budínova 2, CZ-180 81, Prague, Czech Republic; 4 3rd Medical Department – Department of Endocrinology and Metabolism of the First Faculty of Medicine, Charles University in Prague, U nemocnice 1, CZ-128 08, Prague, Czech Republic; 5 Department of Biostatistics, National Institute of Health, Šrobárova 48, CZ-100 42, Prague, Czech Republic

## Abstract

**Introduction:**

Outcomes following bacterial meningitis are significantly improved by adjunctive treatment with corticosteroids. However, little is known about the levels and significance of intrathecal endogenous cortisol. The aim of this study was to assess cortisol as a biological and diagnostic marker in patients with bacterial meningitis.

**Methods:**

Forty-seven consecutive patients with bacterial meningitis and no prior treatment were evaluated. For comparison, a group of 37 patients with aseptic meningitis and a group of 13 healthy control individuals were included.

**Results:**

The mean age of the bacterial meningitis patients was 42 years, and the mean Glasgow Coma Scale, Acute Physiology and Chronic Health Evaluation II, and Sequential Organ Failure Assessment scores on admission were 12, 13 and 4, respectively. Altogether, 40 patients (85%) were admitted to the intensive care unit, with a median (interquartile range) length of stay of 8 (4 to 15) days. A bacterial etiology was confirmed in 35 patients (74%). The median (interquartile range) cortisol concentration in cerebrospinal fluid (CSF) was 133 (59 to 278) nmol/l. CSF cortisol concentrations were positively correlated with serum cortisol levels (*r *= 0.587, *P *< 0.001). Furthermore, CSF cortisol levels correlated with Acute Physiology and Chronic Health Evaluation II score (*r *= 0.763, *P *< 0.001), Sequential Organ Failure Assessment score (*r *= 0.650, *P *< 0.001), Glasgow Coma Scale score (*r *= -0.547, *P *< 0.001) and CSF lactate levels (*r *= 0.734, *P *< 0.001). CSF cortisol was only weakly associated with intrathecal levels of IL-6 (*r *= 0.331, *P = *0.02) and IL-8 (*r *= 0.296, *P *< 0.05). CSF cortisol levels in bacterial and aseptic meningitis significantly differed (*P *< 0.001). The CSF cortisol concentration of 46.1 nmol/l was found to be the optimal cutoff value for diagnosis of bacterial meningitis.

**Conclusion:**

CSF cortisol levels in patients with bacterial meningitis are highly elevated and correlate with disease severity. Moreover, our findings also suggest that intrathecal cortisol may serve as a valuable marker in discriminating between bacterial and aseptic meningitis.

## Introduction

Bacterial meningitis represents a serious disease that is associated with significant morbidity and mortality. Outcomes of bacterial meningitis has remained stable since the advent of antibiotics, with the case fatality being as high as 25% [1]. Furthermore, long-term sequelae such as hearing loss, palsies and personality changes affect approximately 40% of survivors [2]. Early antibiotic therapy is crucial for optimizing the outcome of bacterial meningitis. Therefore, it is important to distinguish bacterial meningitis from aseptic meningitis during the acute phase of the disease, when clinical symptoms are often similar. Current microbiological tests are highly specific, but they lack sufficient sensitivity [3]. Use of various biological markers in blood (C-reactive protein, white blood cell count [WBC], and procalcitonin) or cerebrospinal fluid (CSF; for instance, protein, glucose, WBC, lactate, inflammatory cytokines and combinations thereof) has been suggested to improve sensitivity in determining the aetiological diagnosis [4-8]. However, a sensitive laboratory test that is easy to perform is still required, so that all patients with bacterial meningitis can be identified reliably on admission.

It has been suggested that poor outcomes following bacterial meningitis are significantly influenced by exaggerated immune responses in the brain. The inflammatory brain injury has been associated with overproduction of reactive nitrogen species and tumour necrosis factor (TNF)-α in the intrathecal compartment [9]. Because proinflammatory responses play an important role in the pathogenesis of bacterial meningitis, their modulation may be an important component in the disease management (for review, see the report by Tauber and Moser [10]). Clinical trials have demonstrated that corticosteroids have efficacy in the treatment of bacterial meningitis caused by *Haemophilus influenzae *in children [11]. Recently, a beneficial effect of systemic administration of dexamethasone was documented in adults with bacterial meningitis caused by *Streptococcus pneumoniae *[12].

Although it is known that exogenous corticosteroids can improve the outcome of bacterial meningitis, less is known about the role played by important endogenous anti-inflammatory mediators, such as cortisol and IL-10, in CSF during the course of bacterial meningitis. It is assumed that high levels of IL-10, as were observed in CSF from children with bacterial meningitis, can suppress the intensity of intrathecal inflammation and limit its deleterious effects [13]. Although cortisol has effects similar to those of IL-10, no study of this hormone in the intrathecal compartment during bacterial meningitis has yet been reported in the literature. In contrast, elevated serum cortisol levels have been detected in several studies conducted in paediatric patients with a complicated course of bacterial meningitis [14,15]. Moreover, unstimulated high cortisol levels in serum correlate with an unfavourable outcome of sepsis [16]. However, whether cortisol concentrations are also increased in CSF during bacterial meningitis and whether intrathecal levels of this hormone have prognostic value are not known.

The aim of our study was therefore to evaluate cortisol levels, both in CSF and serum, in the initial phase of bacterial meningitis, and to assess their correlation with inflammatory cytokines as well as routinely examined laboratory parameters. Also, we evaluated relationships between these mediators and the severity of bacterial meningitis, as determined using the Glasgow Coma Scale (GSC), the Acute Physiology and Chronic Health Evaluation (APACHE) II and the Sequential Organ Failure Assessment (SOFA). We also tested whether CSF cortisol levels may correlate with long-term outcome of bacterial meningitis, which was assessed using the Glasgow Outcome Score (GOS). Finally, we tested whether CSF cortisol could be used as a sensitive marker of bacterial meningitis, facilitating distinction of acute bacterial meningitis from aseptic meningitis on admission.

## Materials and methods

### Patients

This prospective study was conducted, in accordance with the Declaration of Helsinki, once approval had been obtained from the local ethics committee, during the period from December 2002 to December 2005. Because we used only leftovers from clinical specimens, the committee waived the need for informed consent. During the study period, 56 patients presenting with suspected bacterial meningitis (in whom this was subsequently confirmed) were admitted to the infectious disease department of a tertiary care hospital. Nine patients were excluded for the following reasons: antibiotic treatment before admission (*n *= 3), administration of methylprednisolone before admission (*n *= 4), and diagnostic lumbar puncture performed elsewhere (*n *= 2). Demographic and clinical data for the 47 patients with bacterial meningitis enrolled in the study are presented in Table 1. The inclusion criteria included age 16 years or greater, duration of symptoms (fever, headache and meningeal irritation) under 72 hours and lumbar puncture performed upon admission to the hospital. A bacterial aetiology disease was confirmed by positive bacterial CSF or blood cultures. In some patients, the aetiology was confirmed by detection bacterial DNA in CSF or peripheral blood using real-time polymerase chain reaction [17].

**Table 1:** Demographic and clinical data of 47 patients with bacterial meningitis

Parameter	Bacterial meningitis patients (*n *= 47)
Demographic characteristics	
Sex (male/female)	29/18
Age (years; mean ± SD)	42 ± 19
Duration of symptoms (hours; *n *[%])	
< 12 hours	14
12–23 hours	13
24–48 hours	12
> 48 hours	8
Clinical characteristics	
APACHE II score (mean ± SD)	12.3 ± 8.9
SOFA score (mean ± SD)	4.0 ± 4.1
GOS (mean ± SD)	4.0 ± 1.6
Septic shock (*n *[%])	5 (10)
Outcome (death at day 28; *n *[%])	7 (15)
Length of hospitalization (days; median [range])	19 (1–123)
Length of ICU stay (days; median [range])	9 (1–123)

APACHE, Acute Physiology and Chronic Health Evaluation; GOS, Glasgow Outcome Score; ICU, intensive care unit; SD, standard deviation; SOFA, Sequential Organ Failure Assessment.

For comparison, findings from a previous study conducted in 37 patients with aseptic meningitis were used [18]. Demographic and clinical data for these patients are presented in Table 2. The control group included 13 persons (eight females and five males; mean age 36.7 years, range 21 to 69 years) with headache and back pain in whom central nervous system infection was ruled out (CSF cytology and clinical chemistry were within normal ranges).

**Table 2:** Demographic and clinical data of 37 patients with aseptic meningitis

Parameter	Aseptic meningitis patients (*n *= 37)
Demographic characteristics	
Sex (male/female)	22/15
Age (years; mean ± SD)	38 ± 18
Duration of symptoms (hours; *n *[%])	
< 24 hours	2
24–72 hours	11
> 72 hours	24
Clinical characteristics	
APACHE II score (mean ± SD)	3 ± 3
SOFA score (mean ± SD)	0.2 ± 0.6
Outcome (death at day 28)	0
Aetiology	
Tick-borne encephalitis virus	10
*Borrelia burgdorferi*	6
Enteroviruses	4
Herpetic viruses (HSV-1/CMV/VZV)	3/1/2
Unknown	11

APACHE, Acute Physiology and Chronic Health Evaluation; CMV, cytomegalovirus; HSV, herpes simplex virus; SD, standard deviation; SOFA, Sequential Organ Failure Assessment; VZV, varicella zoster virus.

### Cerebrospinal fluid and serum sample collection

CSF samples were collected in polystyrene tubes closed with screw-caps (Sarstedt AG, Nümbrecht, Germany). Venous blood was collected into S-Monovette^® ^(Sarstedt AG) with serum separation gel in order to separate blood serum. For glucose and blood count determination, blood was drawn into S-Monovette^® ^tubes with K_3_-EDTA. All samples were centrifuged immediately after the collection, aliquoted and stored at -80°C until further analyses were conducted.

### Cytology and clinical chemistry of cerebrospinal fluid

Leucocyte numbers were determined using a Fuchs-Rosenthal counting chamber (Fein Optik, Jena, Germany) after staining with crystal violet (0.2%) and lysis of erythrocytes with 4% acetic acid. Absolute numbers of mononuclear and segmented cells were determined using the counting chamber. CSF concentrations of glucose, lactate and protein were defined colorimetrically using an automated clinical chemistry analyzer (Vitron™; Ortho Clinical Diagnostics, Inc., Rochester, NY, USA).

### White blood cell count and serum C-reactive protein levels

WBC counts were determined using clinical analyzer Coulter STKS (Coulter Electronics Inc., Miami, FL, USA). Serum C-reactive protein levels were measured using a nephelometer (Behring, Vienna, Austria) using a set Latex CRP Mono (Behring), with normal range between 0 and 8 mg/ml.

### Analysis of cytokines in cerebrospinal fluid and serum

Concentrations of IL-1β, IL-6, IL-8, IL-10, IL-12 and TNF-α in CSF and serum (only in patients with bacterial meningitis) were analyzed using a cytometric bead array kit (BD™ Cytometric Bead Array – Human Inflammatory cytokine kit) and with a three-colour flow cytometer FACSCalibur™ (both BD Biosciences, San Jose, CA, USA). The detection limit for all cytokines was 20 pg/ml.

### Analysis of cortisol in cerebrospinal fluid and serum

The concentration of total cortisol was determined by radioimmunometric assay, using a commercial DSL-2000 kit (Diagnostic Systems Laboratories, Webster, TX, USA). The detection limit for cortisol was 5 nmol/l. The intra-assay and interassay coefficients of variation were measured using patient serum samples and were 5% and 10%, respectively, in all tests.

### Statistical analyses

Statistical analyses were performed using SPSS software™ by a certified biomedical statistician. Data are presented as mean (standard deviation) or as median (interquartile range). Levels that were undetectable were assigned a value equal to the lower limit of detection for the assay. The differences between variables in CSF and serum were analyzed using the Mann-Whitney rank sum test. Differences in analyzed parameters between groups were tested by one-way analysis of variance. The analyses consisted of two-tailed tests with an α level below 0.05. Spearman's correlation test was employed to determine whether a correlation existed between clinical and laboratory parameters. Receiver operating characteristic (ROC) curves, which represent the probability that a test will yield false-positive results, were drawn to determine the optimal cutoff value of CSF cortisol for discriminating bacterial meningitis from aseptic meningitis and controls. The area under the curve was also evaluated.

## Results

### Clinical course and aetiology of bacterial meningitis

Four bacterial meningitis patients presented with septic shock on admission. Favourable outcomes of bacterial meningitis were observed in 36 patients (77%), and seven patients (15%) succumbed to bacterial meningitis within 28 days after admission. Moreover, four patients (8%) exhibited severe neurological sequelae by day 28, and surgery was necessary in six patients after they had completed the antibiotic regimen for bacterial meningitis. The reasons for the surgery were spondylodiscitis (*n *= 3), a communication between sinuses and intracranial space (*n *= 1), brain abscess (*n *= 1) and abdominal surgery (*n *= 1). Altogether, 40 patients (85%) were admitted to the intensive care unit, with an median (interquartile range) length of stay 8 (4 to 15) days.

The bacterial aetiology of bacterial meningitis was confirmed in 35 patients (74%). Out of these 35 cases of bacterial meningitis, 14 (40%) were caused by *Neisseria meningitidis *and 11 cases (31%) were due to *Streptococcus pneumoniae*. Other aetiological agents identified included *Escherichia coli *(*n *= 3), *Staphylococcus aureus *(*n *= 2), *Listeria monocytogenes *(*n *= 2), *Streptococcus bovis *(*n *= 1), *Streptococcus haemolyticus *(*n *= 1) and *Haemophilus influenzae *(*n *= 1).

### Cytology and chemistry of cerebrospinal fluid

Routine cytological and clinical chemistry parameters in serum and CSF in the bacterial meningitis group are summarized in Table 3.

**Table 3:** Cytological and clinical chemistry parameters in blood and CSF in patients with bacterial meningitis

Parameters	Bacterial meningitis patients (*n *= 47)	Normal ranges
Blood		
WBC count (cells/mm^3^)	15,800 (11,825–20,525)	4,000–10,000
CRP (mg/l)	226 (101–308)	0–8
CSF		
WBC count (cells/mm^3^)	3,072 (700–7,443)	< 5
Neutrophil count (cells/mm^3^)	3,030 (654–7,443)	0
Protein (g/l)	3.0 (2.3–6.3)	0.4–0.6
Glucose (mmol/l)	1.4 (0.5–2.98)	2.2–3.3
CSF/serum glucose ratio	0.23 (0.06–0.38)	> 0.45
Lactate (mmol/l)	8.1 (5.6–12.1)	0.9–3.0

Data are expressed as median (interquartile range). CRP, C-reactive protein; CSF, cerebrospinal fluid; WBC, white blood cell.

### Cortisol and cytokines in cerebrospinal fluid and serum

**Figure 1:**
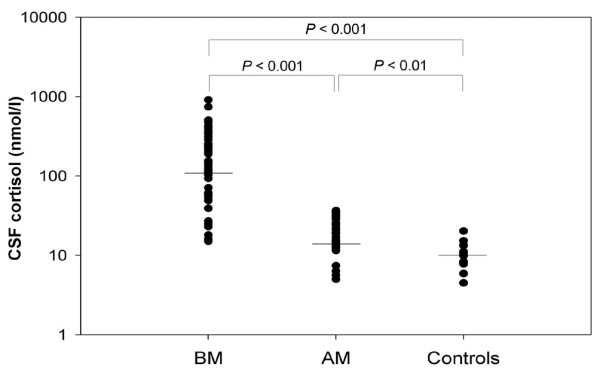
Comparison of cortisol levels in CSF between bacterial and aseptic meningitis and control. Solid lines denote median values. One-way analysis of variance was used. AM, aseptic meningitis; BM, bacterial meningitis; CSF, cerebrospinal fluid.

**Figure 2:**
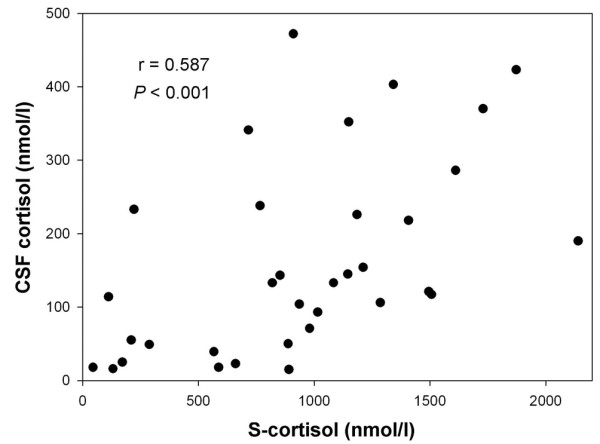
Correlation between CSF and serum cortisol levels. Spearman's correlation test was used. CSF, cerebrospinal fluid; S, serum.

Cortisol and cytokine CSF concentrations in all groups and levels of statistical significance are summarized in Table 4. The comparison of CSF cortisol concentrations between groups is shown in Figure 1. The mean serum cortisol in the bacterial meningitis group was 939 ± 534 nmol/l. Moreover, serum cortisol correlated positively with CSF cortisol concentrations, as shown in Figure 2 (*r *= 0.587, *P *< 0.001).

**Table 4:** Comparison of cortisol and cytokine levels in CSF between bacterial and aseptic meningitis and controls

Parameter in CSF	Bacterial meningitis patients (*n *= 47)	Aseptic meningitis patients (*n *= 37)	Control individuals (*n *= 13)	*P*^a^
TNF-α (pg/ml)	245 (20–4,978)	< 20	< 20	0.001
IL-1β (pg/ml)	254 (20–1,239)	N/A	< 20	N/A
IL-6 (pg/ml)	187,245 (75,275–312,289)	157 (38–410)	< 20	0.001
IL-8 (pg/ml)	16,830 (4,776–133,236)	130 (54–321)	< 20	0.001
IL-10 (pg/ml)	260 (20–1,153)	71 (61–116)	< 20	0.001
Cortisol (nmol/l)	133 (59–278)	17 (13–28)	10 (8–12)	0.001

Data are presented as median (interquartile range). ^a^Bacterial meningitis versus aseptic meningitis (Mann-Whitney *U*-test). CSF, cerebrospinal fluid; ICU, intensive care unit; IL, interleukin; N/A, not available; TNF, tumour necrosis factor.

### Correlation of cerebrospinal fluid cortisol levels and other parameters

**Figure 3:**
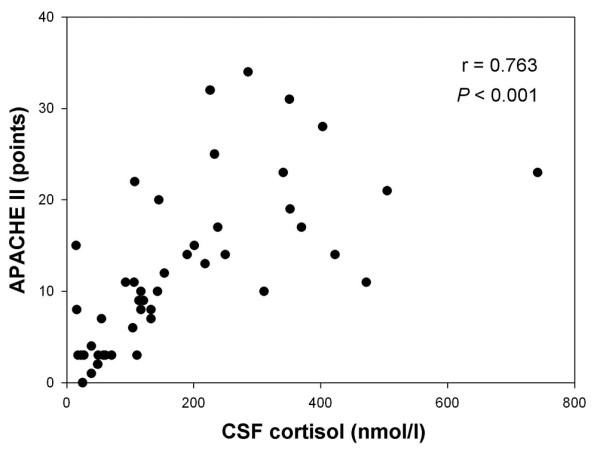
Correlation between CSF cortisol and APACHE II score in bacterial meningitis. Spearman's correlation test was used. APACHE, Acute Physiology and Chronic Health Evaluation; CSF, cerebrospinal fluid.

**Figure 4:**
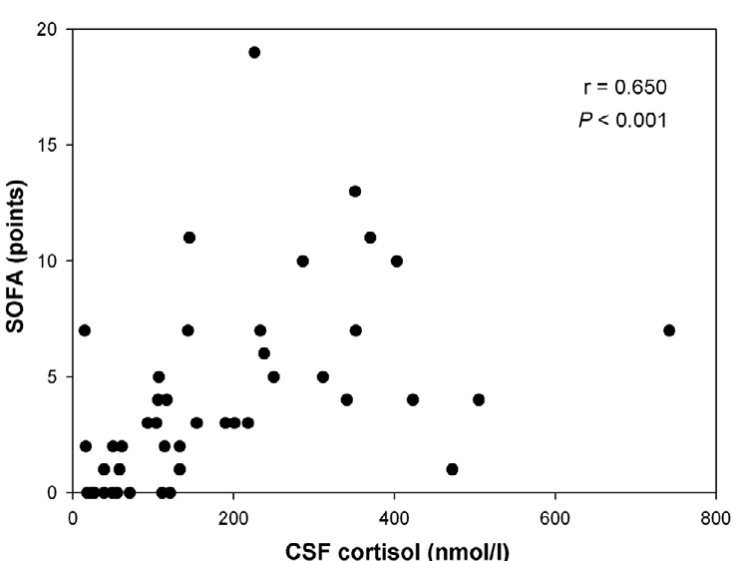
Correlation between CSF cortisol concentration and SOFA score in bacterial meningitis. Spearman's correlation test was used. CSF, cerebrospinal fluid; SOFA, Sequential Organ Failure Assessment.

The primary aim was to assess the relationship between the severity of bacterial meningitis and CSF cortisol as well as CSF levels of inflammatory cytokines. CSF cortisol concentration exhibited a positive correlation with APACHE II score (*r *= 0.763, *P *< 0.001; Figure 3) and SOFA score (*r *= 0.650, *P *< 0.001; Figure 4), and a negative relationship with GCS score (*r *= -0.547, *P *< 0.001). Also, we found a correlation between GOS score and CSF cortisol (*r *= -0.276, *P *= 0.06). Of six cytokines evaluated in CSF, only moderate correlations with CSF cortisol were found for IL-6 (*r *= 0.331, *P = *0.02) and IL-8 (*r *= 0.296, *P *< 0.05). Additionally, there was no correlation between all evaluated cytokines and clinical scores.

Further *post hoc *analyses identified correlations between CSF cortisol and CSF lactate (*r *= 0.734, *P *< 0.001) and protein (*r *= 0.534, *P *< 0.001). Also, associations were detected between CSF level of IL-6 and lactate (*r *= 0.668, *P *< 0.001), protein (*r *= 0.701, *P *< 0.001), IL-8 (*r *= 0.451, *P *< 0.001) and WBC count in CSF (*r *= 0.475, *P *< 0.001). Finally, intrathecal levels of IL-8 (*r *= 0.739, *P *< 0.001) and IL-10 (*r *= 0.444, *P = *0.002) correlated positively with CSF concentrations of TNF-α.

### Correlation of serum cortisol concentration and other parameters

The initial aim of the study was to evaluate the association between serum cortisol, cytokines and severity of bacterial meningitis. As was expected, serum cortisol exhibited a positive correlation with APACHE II score (*r *= 0.399, *P *= 0.014) and SOFA score (*r *= 0.394, *P *= 0.016). Of the cytokines evaluated, IL-8 correlated with APACHE II, SOFA and GCS scores (*r *= 0.554 [*P *= 0.001], *r *= 0.519 [*P *= 0.002] and *r *= -0.421 [*P *= 0.02], respectively), as did IL-6 (*r *= 0.386 [*P *= 0.03], *r *= 0.389 [*P *= 0.03] and *r *= -0.401 [*P *= 0.02], respectively). No correlation was found between other serum cytokines (IL-1β, IL-10, IL-12 and TNF-α) and severity of bacterial meningitis. Also, analyses revealed that serum cortisol levels correlated positively with IL-6 (*r *= 0.696, *P *< 0.001), IL-10 (*r *= 0.501, *P = *0.001) and IL-8 (*r *= 0.612, *P *< 0.001).

In addition, further *post hoc *analyses demonstrated that IL-10 levels were positively associated with IL-6 (*r *= 0.620, *P *< 0.001) and IL-8 (*r *= 0.460, *P *= 0.002) in serum. Finally, a relationship was observed between serum concentrations of IL-6 and IL-8 (*r *= 0.629, *P *< 0.001).

### Evaluation of cerebrospinal fluid cortisol as a marker for discriminating between acute bacterial meningitis and acute aseptic meningitis

**Figure 5:**
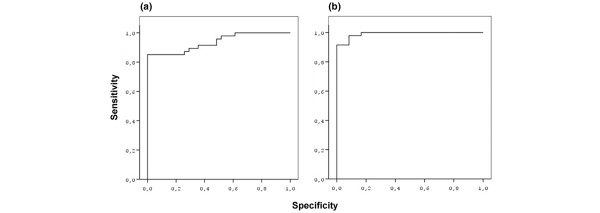
ROC curves for cortisol in CSF. The ROC curves were calculated for the discrimination of **(a) **bacterial from aseptic meningitis and **(b) **bacterial meningitis from healthy controls. CSF, cerebrospinal fluid; ROC, receiver operating characteristic.

The levels of CSF cortisol in bacterial meningitis and aseptic meningitis differed significantly (*P *< 0.001; Table 3). After setting the threshold of 46.1 nmol/l using ROC analysis, we identified a specificity of 100% and a sensitivity of 82% for the CSF cortisol test for discriminating bacterial meningitis patients from aseptic meningitis patients, with an AUC of 0.94 (Figure 5a). The optimal threshold for CSF cortisol for discriminating between bacterial meningitis patients and control individuals was found to be 12.9 nmol/l, for which the sensitivity and specificity were both 100%, and the AUC was 0.99 (Figure 5b).

## Discussion

In this study we hypothesized that CSF cortisol levels would be elevated in patients with bacterial meningitis and that this increase might correlate with disease severity. Also, we aimed to identify a level of CSF cortisol that could serve as a marker of bacterial meningitis.

In accordance with our assumption, CSF cortisol concentrations were significantly elevated in patients with bacterial meningitis as compared with concentrations in patients with aseptic meningitis as well as in healthy control individuals. We report here, for the first time, the cutoff value of CSF cortisol that separates bacterial meningitis from aseptic meningitis on admission, with good sensitivity and specificity. Moreover, we found strong correlations between high CSF cortisol and elevated APACHE II, SOFA and GCS scores in the 47 patients with acute bacterial meningitis. Similar associations were observed for serum cortisol. As expected, our results revealed highly elevated concentrations of the majority of detected cytokines in CSF, and confirmed the previously described compartmentalization of the inflammatory response in the subarachnoideal space as compared with peripheral blood [19].

Interestingly, the increased CSF cortisol levels exhibited a strong correlation with the severity of bacterial meningitis, whereas highly elevated intrathecal cytokine concentrations – especially IL-6 and IL-8 – exhibited no relationship with clinical scores. Because this is the first study of CSF cortisol during the acute stage of bacterial meningitis, it raises concerns about the pathophysiological and clinical importance of this hormone. With regard to serum cortisol levels during the course of bacterial meningitis, van Woensel and coworkers [14] reported higher concentrations in patients with meningococcal meningitis than in those with fulminant meningococcal sepsis, which is the most severe form of invasive meningococcal disease. In contrast, we observed a significant relationship between high CSF and serum cortisol levels and a severe course of bacterial meningitis. The difference between our findings and those reported by van Woensel and coworkers might result from the fact that fulminant meningococcal sepsis is associated with a blunted cortisol response, whereas this response is preserved during the course of meningitis [14,20].

Increased CSF cortisol levels have previously been reported in various CNS disorders, such as multiple sclerosis, Alzheimer's disease, depression and post-traumatic stress disorder [21-23]; however, the CSF cortisol concentrations were much higher in our group of patients with bacterial meningitis. These differences are most likely due to the severity of bacterial meningitis, which is associated with systemic inflammation, intense stress response and compromised blood-brain barrier [19]. This is also supported by the results of multivariate analysis (data not shown), which demonstrated similar relationship between serum or CSF cortisol with APACHE II scores in our patients with bacterial meningitis. However, previous studies have documented that CSF cortisol levels cannot be inferred directly from serum levels [24-26]. It was suggested that balance between CSF and blood cortisol levels is controlled by active efflux of the hormone from the brain [27]. Perturbation of this mechanism by inflammation, together with reduced ability of brain cells to metabolize sterol molecules, may lead to a persistent increase in CSF cortisol. Another mechanism that may participate in elevated CSF cortisol levels observed in bacterial meningitis patients could be *de novo *synthesis of cortisol, catalyzed by the brain enzyme 11β-hydroxysteroid dehydrogenase type 1 [28]. Also, traversal of cortisol across the blood-brain barrier is probably promoted by the amount of free (protein unbound) cortisol during sepsis [29]. During critical illness, cortisol-binding globulin and albumin blood levels decrease by about 50%, leading to an increase in biologically active free cortisol. It is likely that the increase in CSF cortisol during the course of bacterial meningitis is mostly due to this free fraction. However, because of bacterial meningitis-induced damage to the blood-brain barrier, cortisol transport via cortisol-binding globulin must also be considered.

Our study in patients with bacterial meningitis demonstrates that the brain is exposed to cortisol levels that are substantially greater than normal. Moreover, the correlation observed between CSF cortisol and GOS in our patients with bacterial meningitis suggest that the high level of intrathecal cortisol could exacerbate bacterial meningitis-related inflammatory brain injury. In a rabbit model of experimentally induced pneumococcal meningitis, Zysk and coworkers [30] reported that dexamethasone can increase neuronal cell death in the hippocampus. On the other hand, in the same study dexamethasone reduced overall neuronal damage. Furthermore, it is worth noting that cortisol may attenuate the inflammatory response causing brain tissue injury [31]. Cortisol can also reduce production of reactive oxygen species from polymorphonuclear cells, which are the most abundant inflammatory cells in CSF during bacterial meningitis [32]. The significant correlation that we identified between CSF levels of cortisol and lactate also raises a question about their relationship. If we assume that CSF lactate is mostly produced by host cells [33], then the association between intrathecal cortisol and lactate levels may indicate that there is an effect of cortisol on brain tissue metabolism.

It has previously been proposed that both inflammatory cytokines and lactate in CSF may represent reliable laboratory markers for discriminating between bacterial meningitis and aseptic meningitis [4]. Our finding of the significant difference in CSF cortisol level between patients with bacterial meningitis and those with aseptic meningitis also suggests that it has diagnostic value. Moreover, we determined the CSF cortisol level that yields 100% specificity and 82% sensitivity in discriminating between bacterial meningitis and aseptic meningitis. In a recent study of 16 diagnostic markers of meningitis [4], only granulocytes, lactate, IL-6 and IL-1β in CSF exhibited similar reliability. Routine laboratory tests for detection of IL-6 and IL-1β are not available in most hospitals. Also, no single CSF test has yet proved to be fully reliable in distinguishing bacterial meningitis from aseptic meningitis so far, and various CSF parameters must be combined. Thus, addition of a new parameter, such as CSF cortisol, to the aforementioned panel of tests to permit rapid aetiological diagnosis in meningitis is desirable.

Certain limitations of our study should be considered. The difference in CSF cortisol levels found between bacterial meningitis and aseptic meningitis patients might partly be influenced by differences in severity between these two central nervous system infections. Also, CSF cortisol levels were detected using the radioimmunoassay method, which is not suitable for use in the clinical setting. Therefore, the value of CSF cortisol as a diagnostic biomarker requires confirmation in a larger, prospective clinical study.

## Conclusion

Intrathecal levels of cortisol, as opposed to serum levels, may represent a valuable biomarker of the severity of bacterial meningitis. Moreover, CSF cortisol may help to discriminate bacterial meningitis from aseptic meningitis reliably. Finally, our findings support the pathophysiological importance of intrathecal cortisol during bacterial meningitis, and further studies are warranted to elucidate the role played by this mediator in brain.

## Key messages

• CSF and serum cortisol levels are markers of the severity of bacterial meningitis.

• CSF cortisol levels are significantly higher in bacterial meningitis than in aseptic meningitis, which may help in differentiating between them.

• The pathophysiological effects of high CSF cortisol concentrations during bacterial meningitis are unclear.

## Abbreviations

APACHE = Acute Physiology and Chronic Health Evaluation; CSF = cerebrospinal fluid; GCS = Glasgow Coma Scale; GOS = Glasgow Outcome Score; IL = interleukin; ROC = receiver operating characteristic, SOFA = Sequential Organ Failure Assessment; TNF = tumour necrosis factor; WBC = white blood cell.

## Competing interests

The authors declare that they have no competing interests.

## Authors' contributions

MH designed and coordinated the study, collected data and drafted the manuscript. OB participated in the design of the study, supervised the laboratory experiments and helped to write the manuscript. OD helped to collect patient samples and data. JH carried out the CBA experiments and helped to collect patient samples and data. ZL carried out the cortisol analysis. BP is a certified statistician and conducted statistical analyses. JP and MH helped to collect patient data. All authors read and approved the final manuscript.
